# Supplementary figures and images for: The role of ER exit sites in maintaining P-body organization and integrity during Drosophila melanogaster oogenesis
Source: EMBO Rep. 2024 Dec 9;26(2):494–520. doi: 10.1038/s44319-024-00344-x (PMC11772875; doi:10.1038/s44319-024-00344-x)

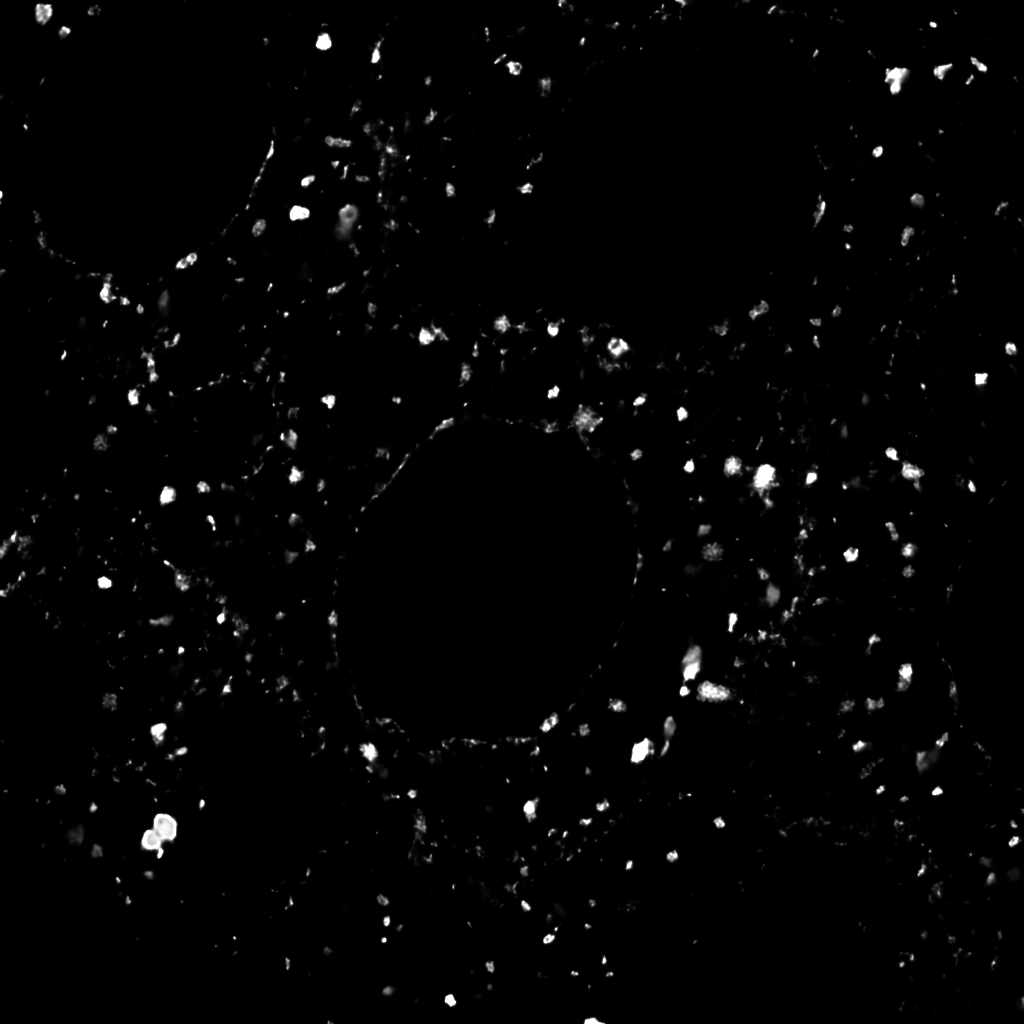

Supplement: Supplementary file 4 — Source data Fig. 1 [file 44319_2024_344_MOESM4_ESM.zip › Source data Fig. 1/1B/1B.tif]

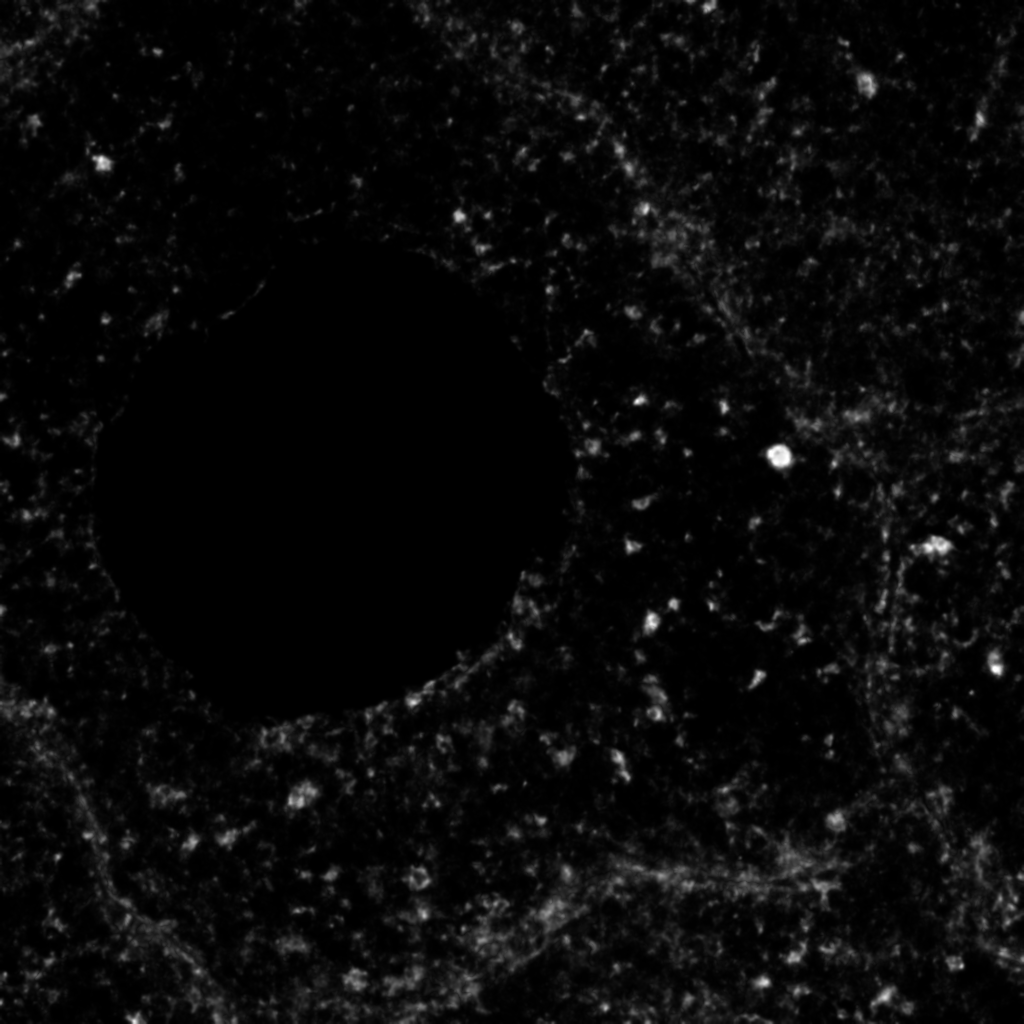

Supplement: Supplementary file 4 — Source data Fig. 1 [file 44319_2024_344_MOESM4_ESM.zip › Source data Fig. 1/1E/1E.tif]

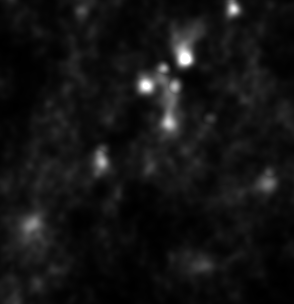

Supplement: Supplementary file 4 — Source data Fig. 1 [file 44319_2024_344_MOESM4_ESM.zip › Source data Fig. 1/1H/1H.tif]

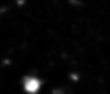

Supplement: Supplementary file 5 — Source data Fig. 2 [file 44319_2024_344_MOESM5_ESM.zip › Source data Fig. 2/2A/2A.tif]

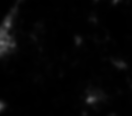

Supplement: Supplementary file 5 — Source data Fig. 2 [file 44319_2024_344_MOESM5_ESM.zip › Source data Fig. 2/2B/2B.tif]

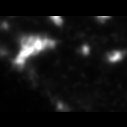

Supplement: Supplementary file 6 — Source data Fig. 3 [file 44319_2024_344_MOESM6_ESM.zip › Source Data Fig. 3/3A/3A.tif]

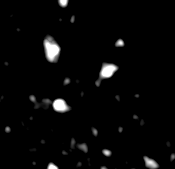

Supplement: Supplementary file 6 — Source data Fig. 3 [file 44319_2024_344_MOESM6_ESM.zip › Source Data Fig. 3/3B/3B.tif]

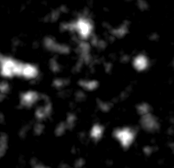

Supplement: Supplementary file 6 — Source data Fig. 3 [file 44319_2024_344_MOESM6_ESM.zip › Source Data Fig. 3/3E/3E.tif]

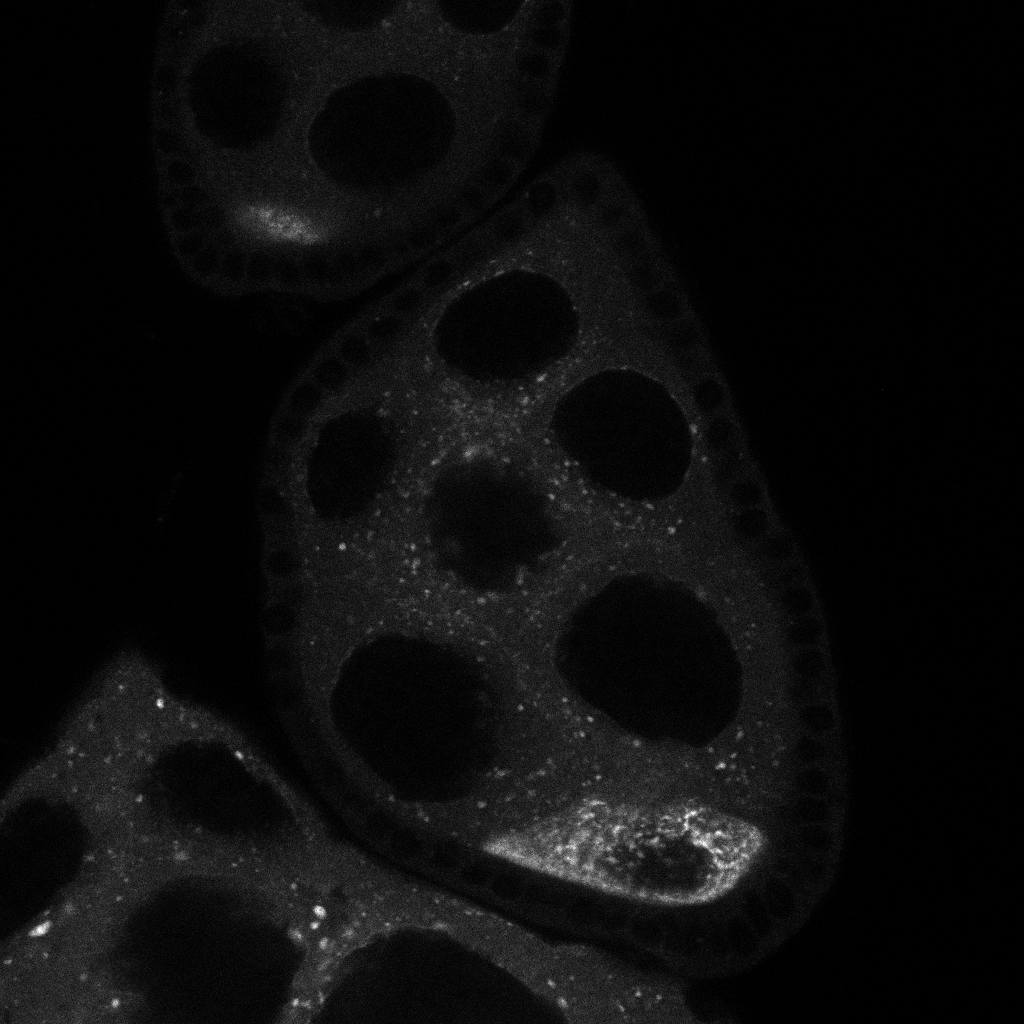

Supplement: Supplementary file 7 — Source data Fig. 4 [file 44319_2024_344_MOESM7_ESM.zip › Source Data Fig. 4/4B/B_Sec23 RNAi.tif]

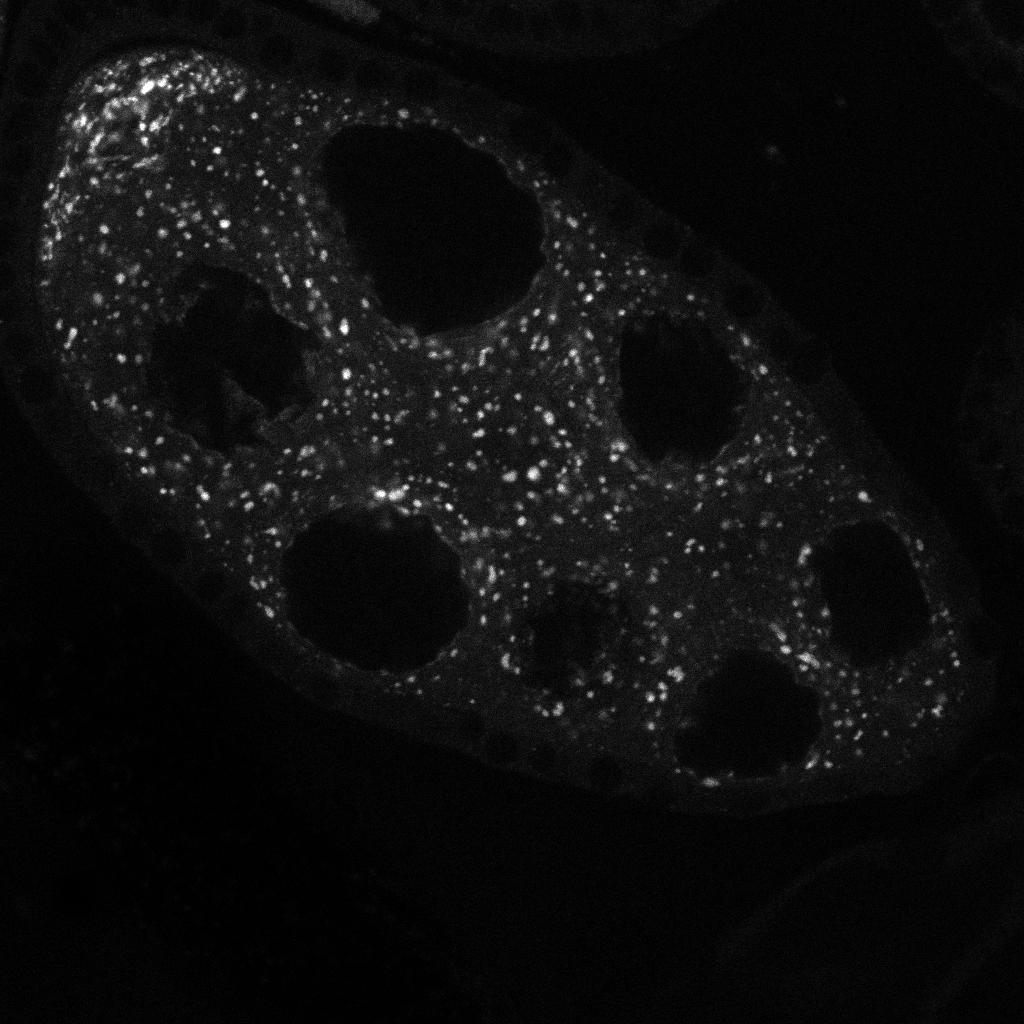

Supplement: Supplementary file 7 — Source data Fig. 4 [file 44319_2024_344_MOESM7_ESM.zip › Source Data Fig. 4/4B/B Sec31 RNAi.tif]

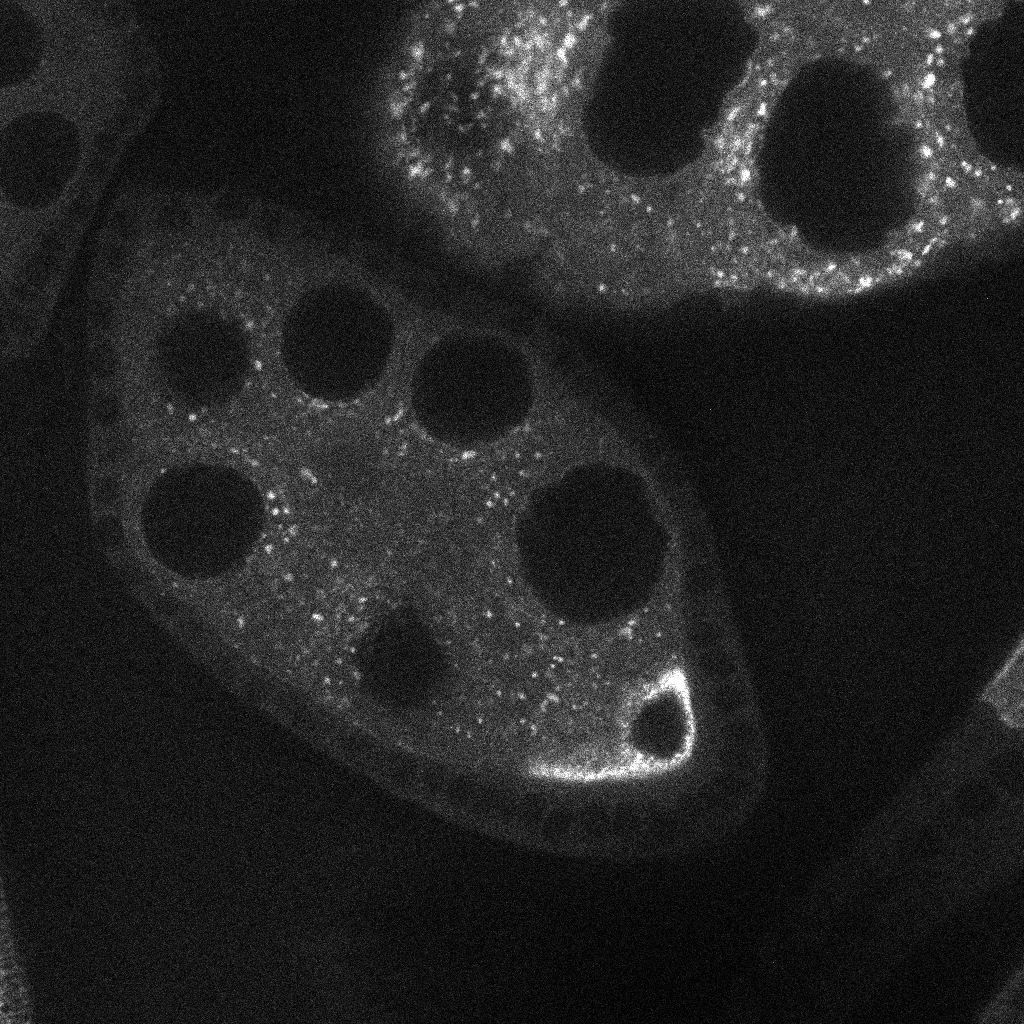

Supplement: Supplementary file 7 — Source data Fig. 4 [file 44319_2024_344_MOESM7_ESM.zip › Source Data Fig. 4/4B/B_mCherry RNAi.tif]

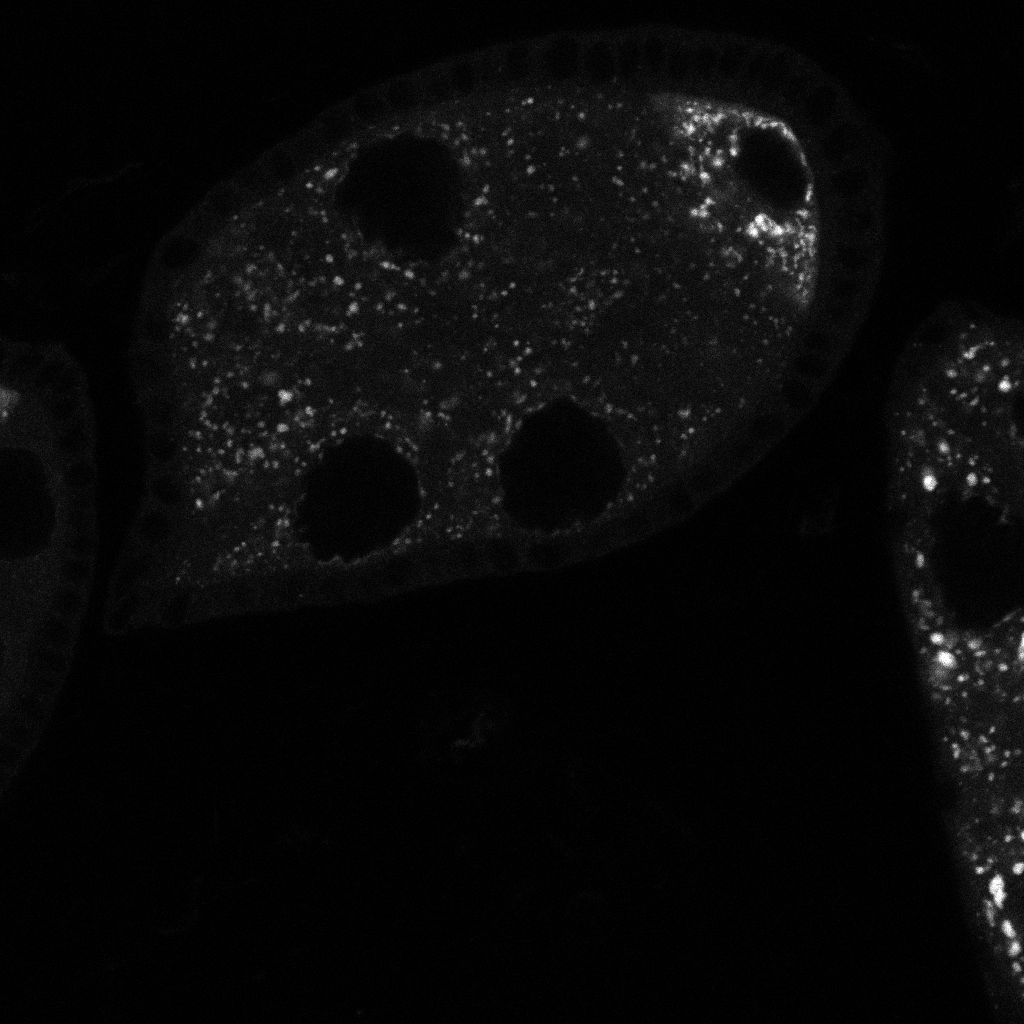

Supplement: Supplementary file 7 — Source data Fig. 4 [file 44319_2024_344_MOESM7_ESM.zip › Source Data Fig. 4/4B/B_Sec13 RNAi.tif]

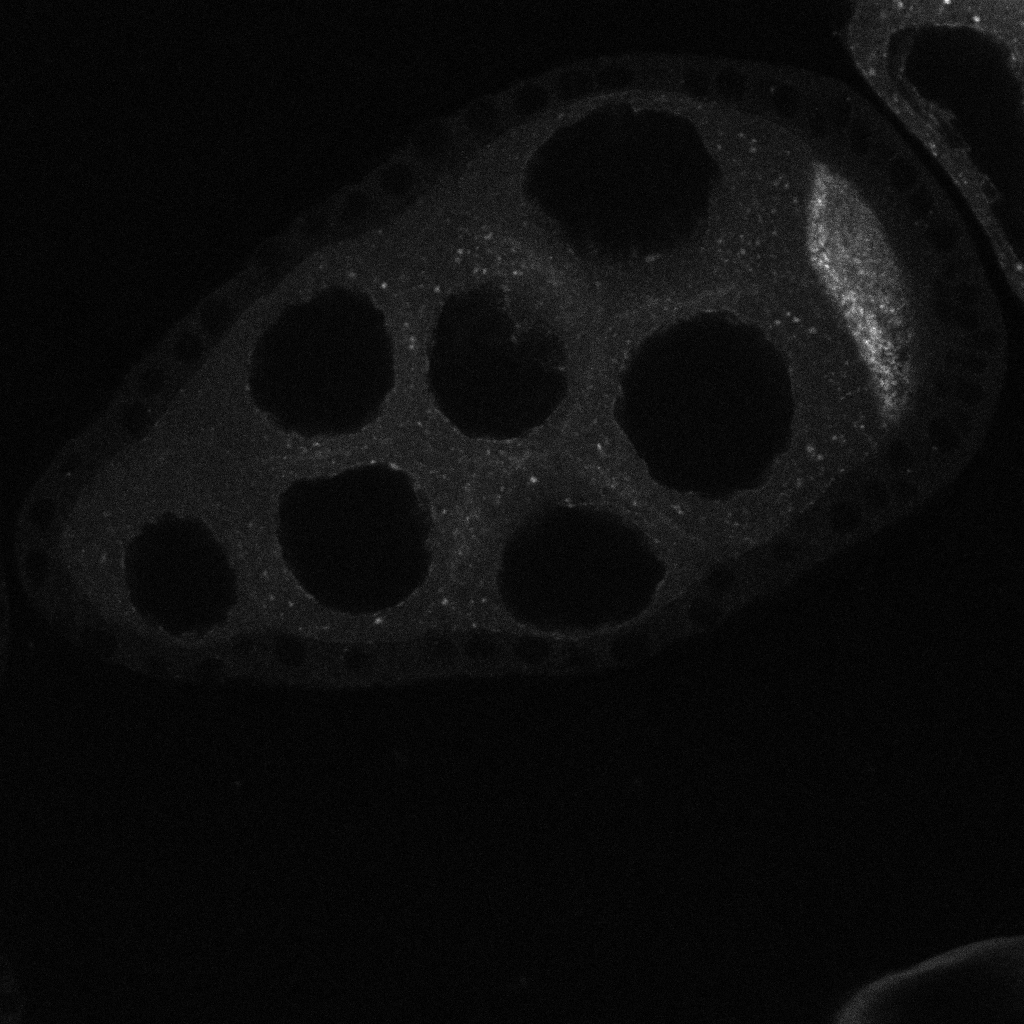

Supplement: Supplementary file 7 — Source data Fig. 4 [file 44319_2024_344_MOESM7_ESM.zip › Source Data Fig. 4/4I/I.tif]

mCherry

Sec16

Sec23

Sec13

Sec31

Cup

Tral-RFP

Tri-methyl  
Histone

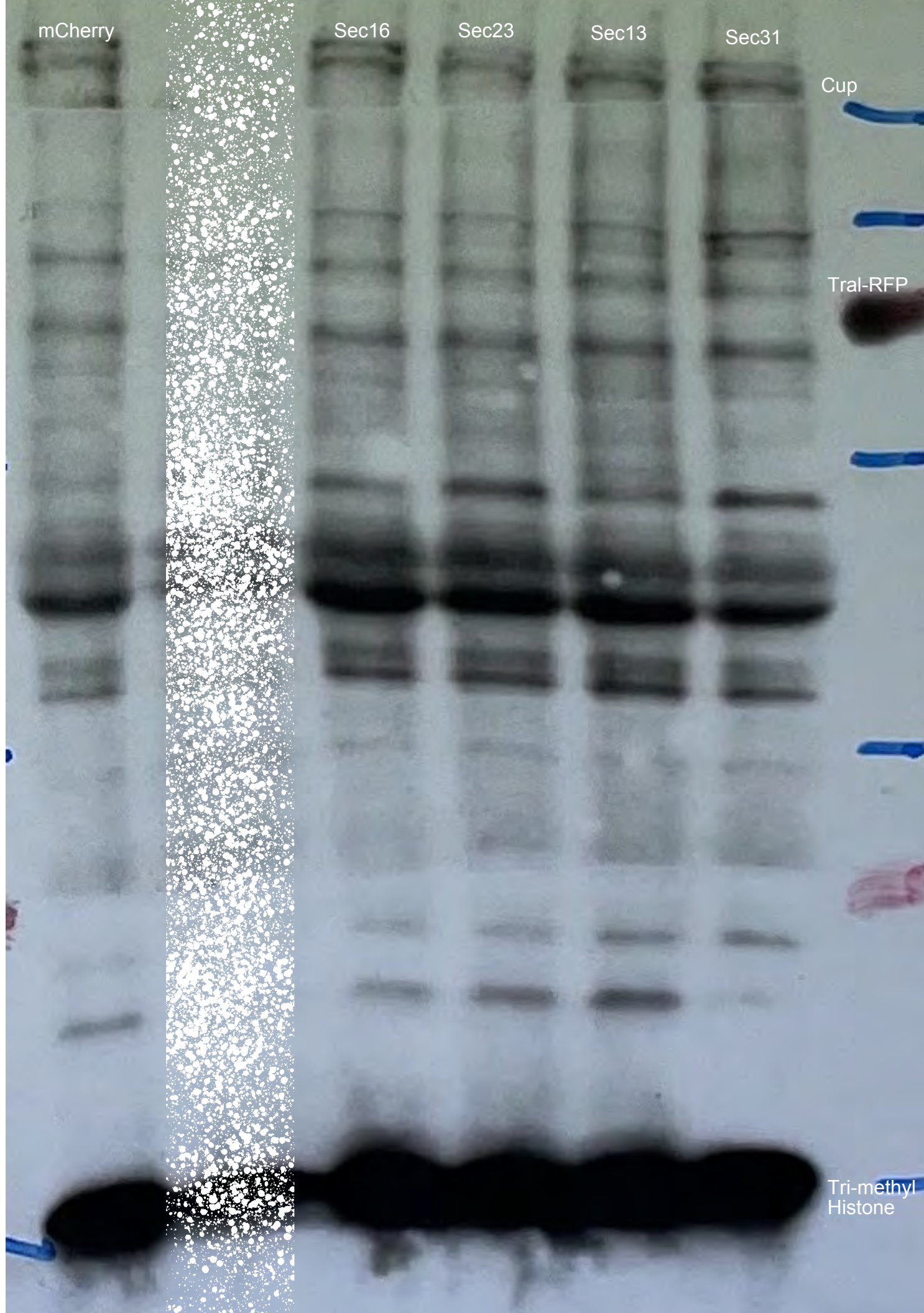

Supplement: Supplementary file 7 — Source data Fig. 4 [file 44319_2024_344_MOESM7_ESM.zip › Source Data Fig. 4/4J/4J.pdf]

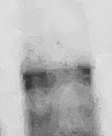

Supplement: Supplementary file 8 — Source data Fig. 5 [file 44319_2024_344_MOESM8_ESM.zip › Source Data Fig. 5/5G/Cup Protein/Cup_sar1RNAi.tif]

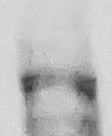

Supplement: Supplementary file 8 — Source data Fig. 5 [file 44319_2024_344_MOESM8_ESM.zip › Source Data Fig. 5/5G/Cup Protein/Cup_mCherryRNAi.tif]

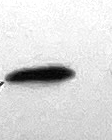

Supplement: Supplementary file 8 — Source data Fig. 5 [file 44319_2024_344_MOESM8_ESM.zip › Source Data Fig. 5/5G/Loading Control/Loading Control sar1RNAi.tif]

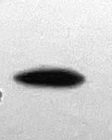

Supplement: Supplementary file 8 — Source data Fig. 5 [file 44319_2024_344_MOESM8_ESM.zip › Source Data Fig. 5/5G/Loading Control/Loading Control mCherryRNAi.tif]

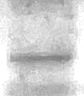

Supplement: Supplementary file 8 — Source data Fig. 5 [file 44319_2024_344_MOESM8_ESM.zip › Source Data Fig. 5/5G/Me31B Protein/Me31B_sar1RNAi.tif]

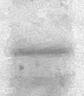

Supplement: Supplementary file 8 — Source data Fig. 5 [file 44319_2024_344_MOESM8_ESM.zip › Source Data Fig. 5/5G/Me31B Protein/Me31B_mCherryRNAi.tif]

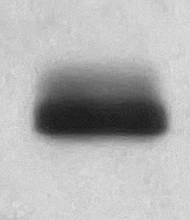

Supplement: Supplementary file 8 — Source data Fig. 5 [file 44319_2024_344_MOESM8_ESM.zip › Source Data Fig. 5/5G/Tral Protein/tral sar1.tif]

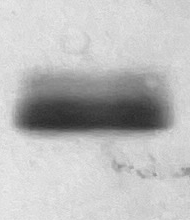

Supplement: Supplementary file 8 — Source data Fig. 5 [file 44319_2024_344_MOESM8_ESM.zip › Source Data Fig. 5/5G/Tral Protein/tral wt.tif]

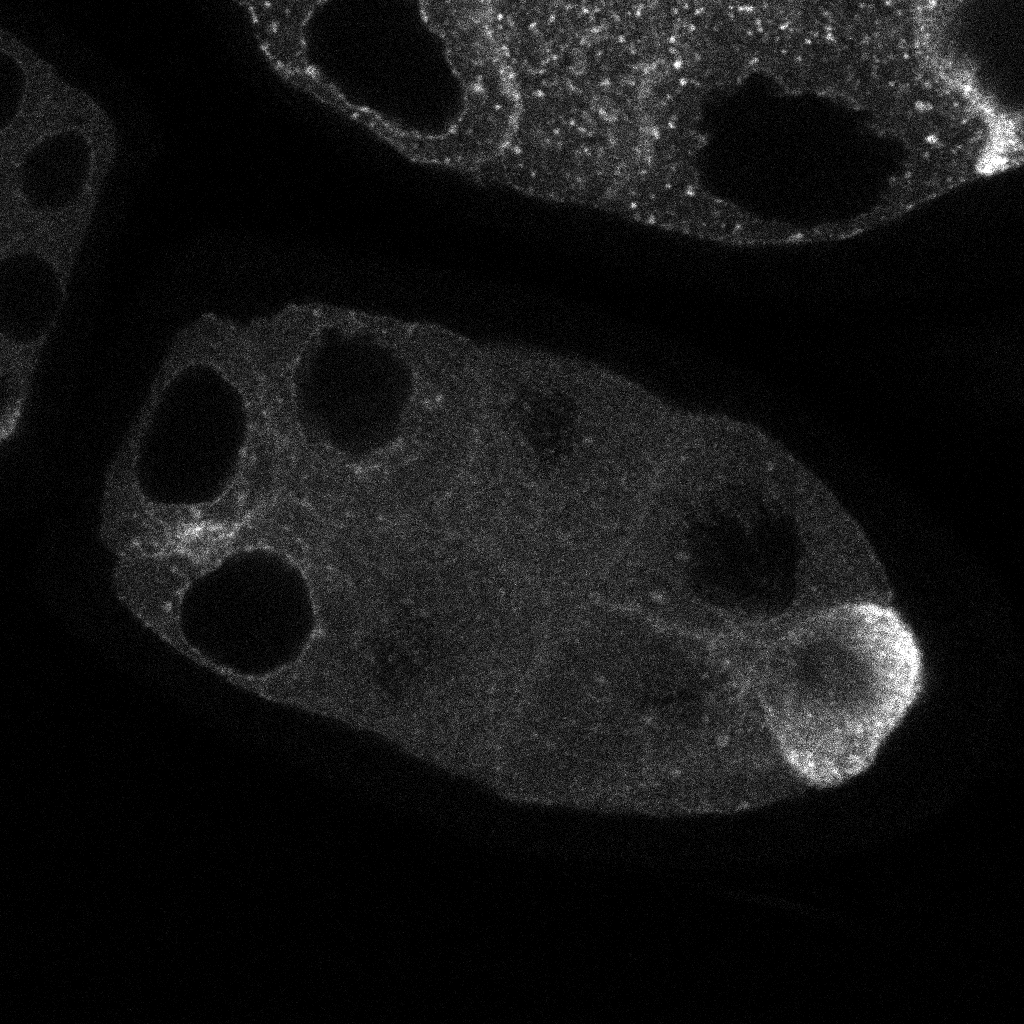

Supplement: Supplementary file 8 — Source data Fig. 5 [file 44319_2024_344_MOESM8_ESM.zip › Source Data Fig. 5/5A/A_sar1RNAi.tif]

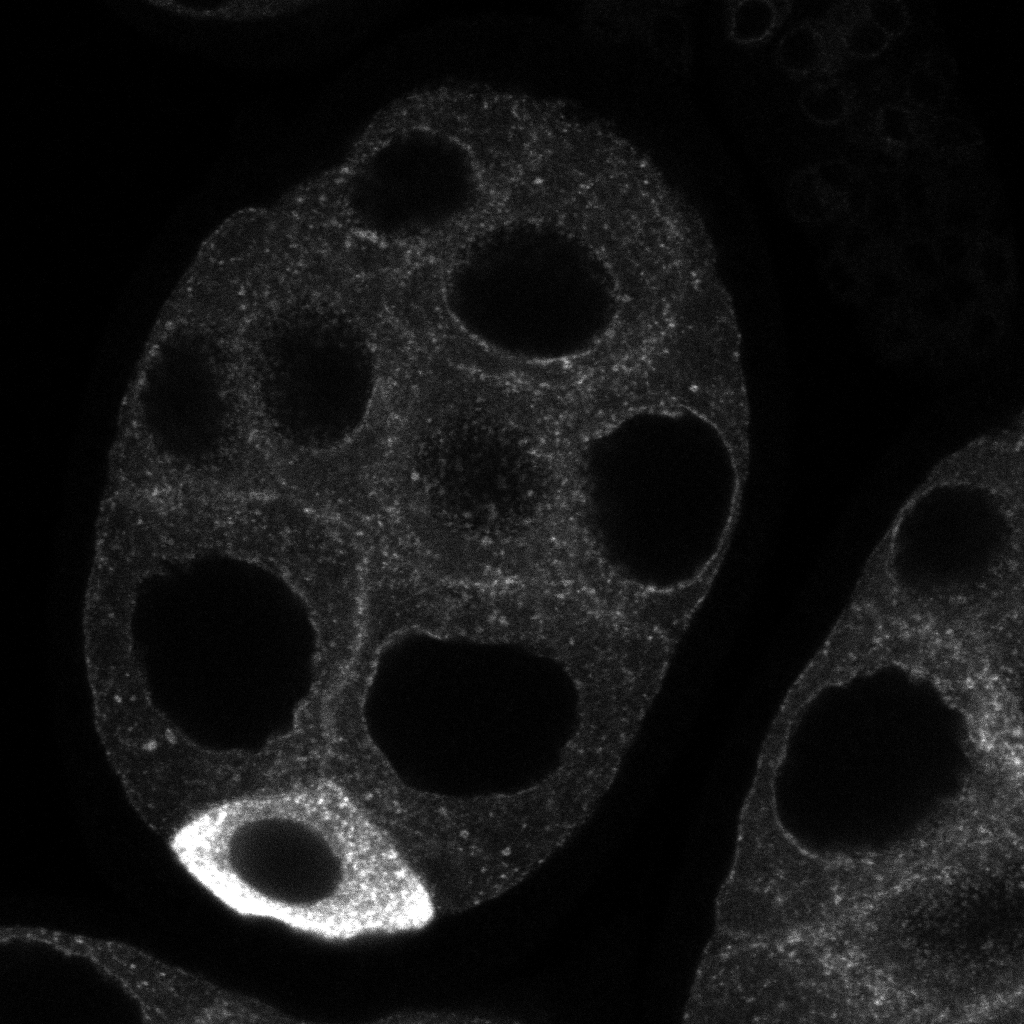

Supplement: Supplementary file 8 — Source data Fig. 5 [file 44319_2024_344_MOESM8_ESM.zip › Source Data Fig. 5/5A/A_mCherryRNAi.tif]

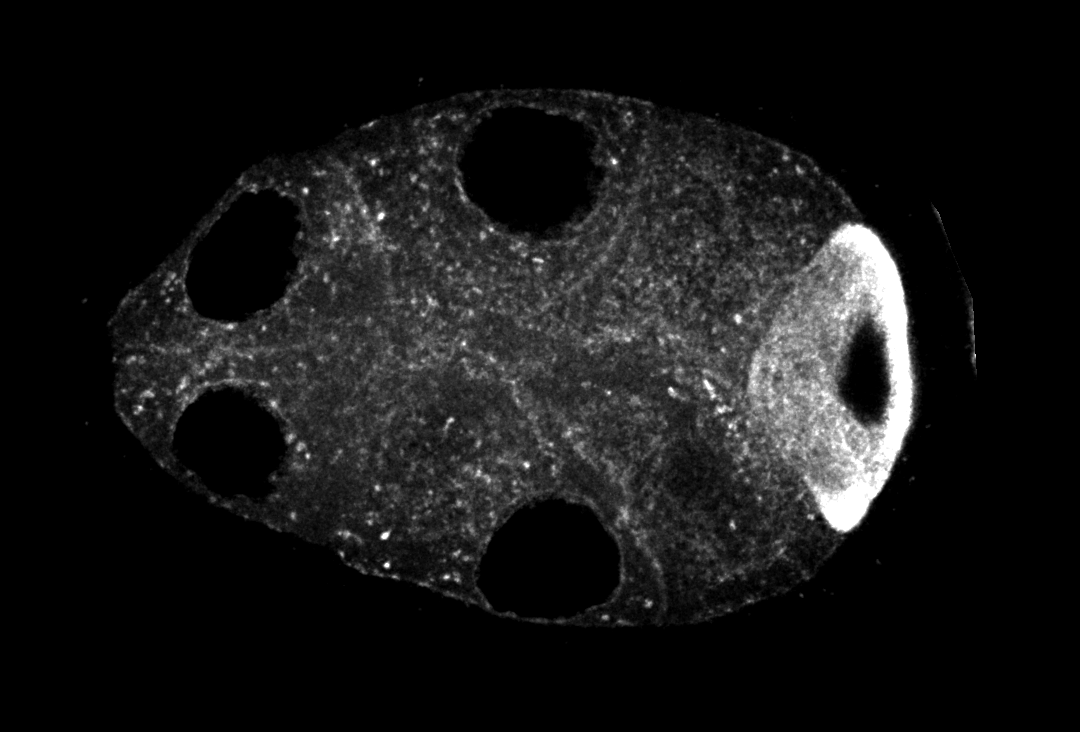

Supplement: Supplementary file 8 — Source data Fig. 5 [file 44319_2024_344_MOESM8_ESM.zip › Source Data Fig. 5/5D/D.tif]

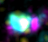

Supplement: Supplementary file 8 — Source data Fig. 5 [file 44319_2024_344_MOESM8_ESM.zip › Source Data Fig. 5/5H/H.tif]

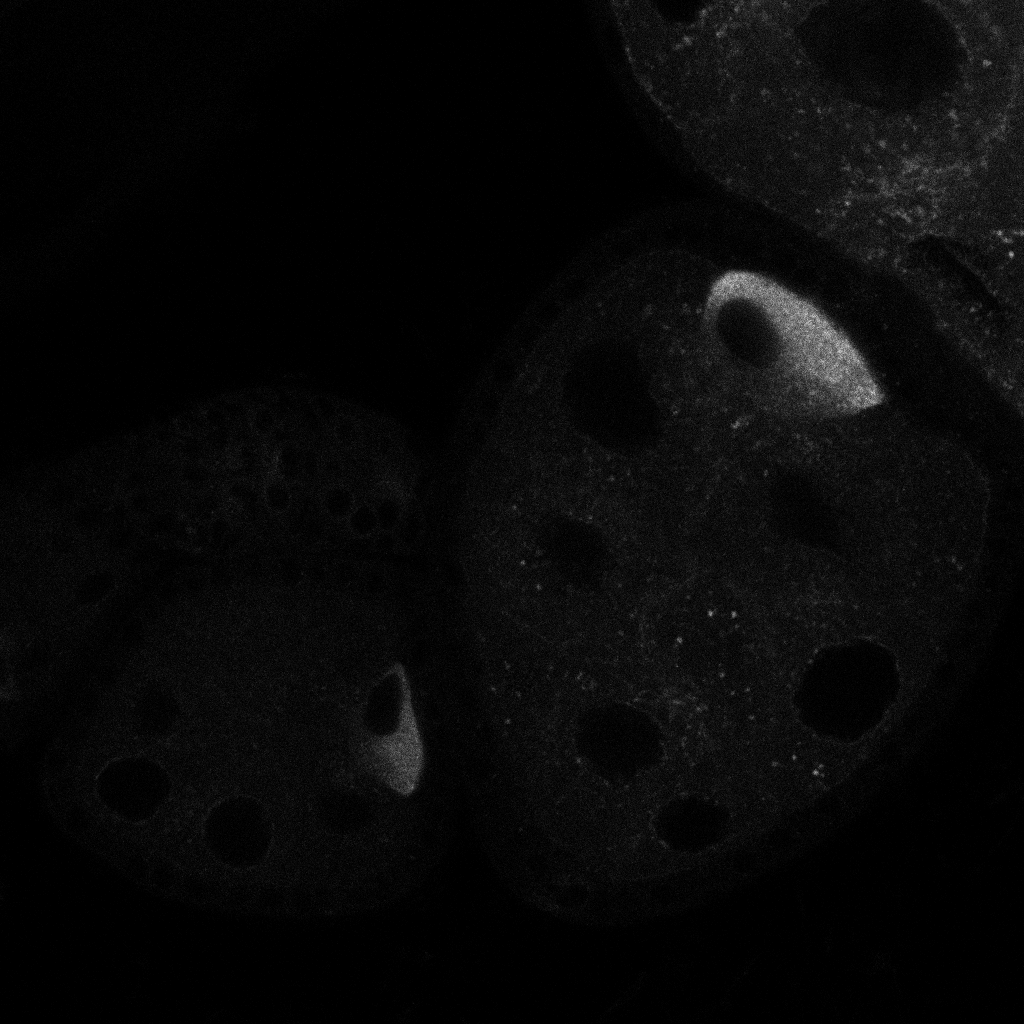

Supplement: Supplementary file 8 — Source data Fig. 5 [file 44319_2024_344_MOESM8_ESM.zip › Source Data Fig. 5/5I/I_mCherryRNAi.tif]

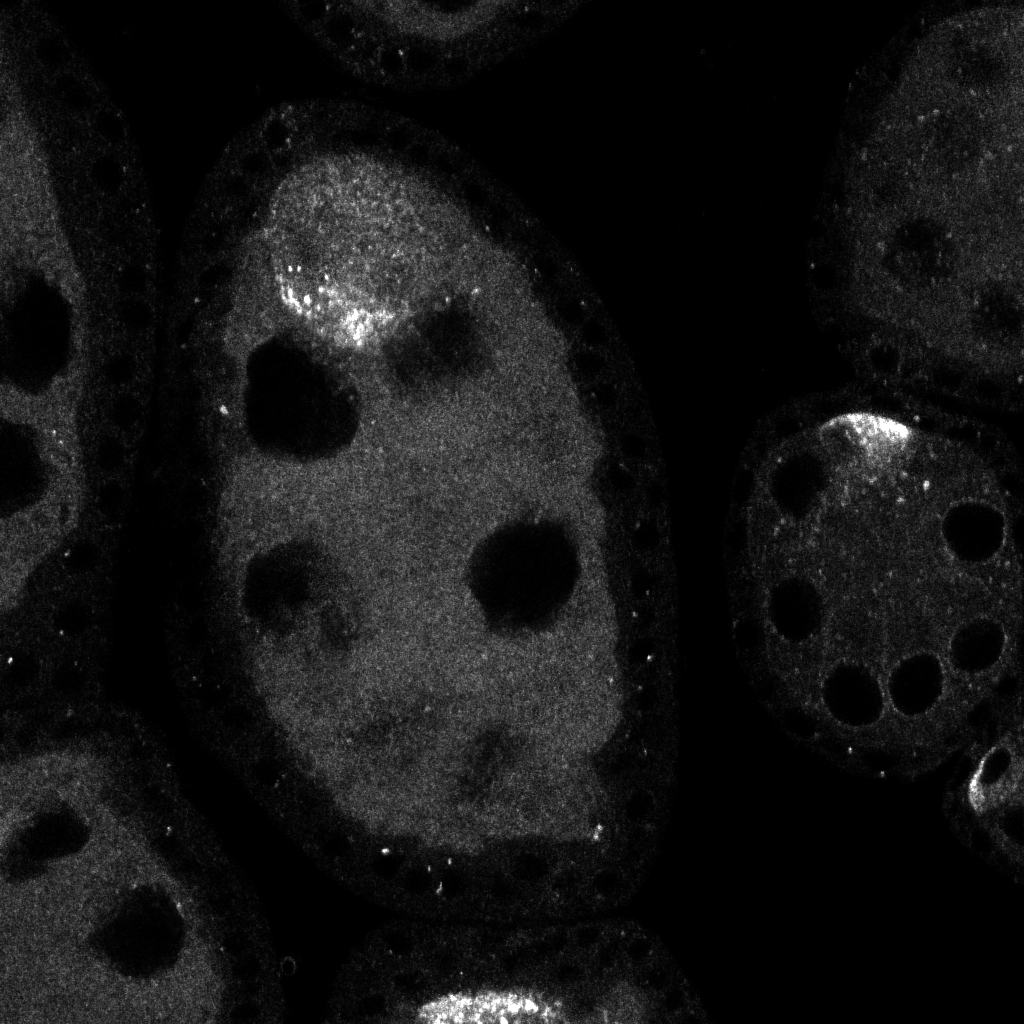

Supplement: Supplementary file 8 — Source data Fig. 5 [file 44319_2024_344_MOESM8_ESM.zip › Source Data Fig. 5/5I/I_sar1RNAi.tif]

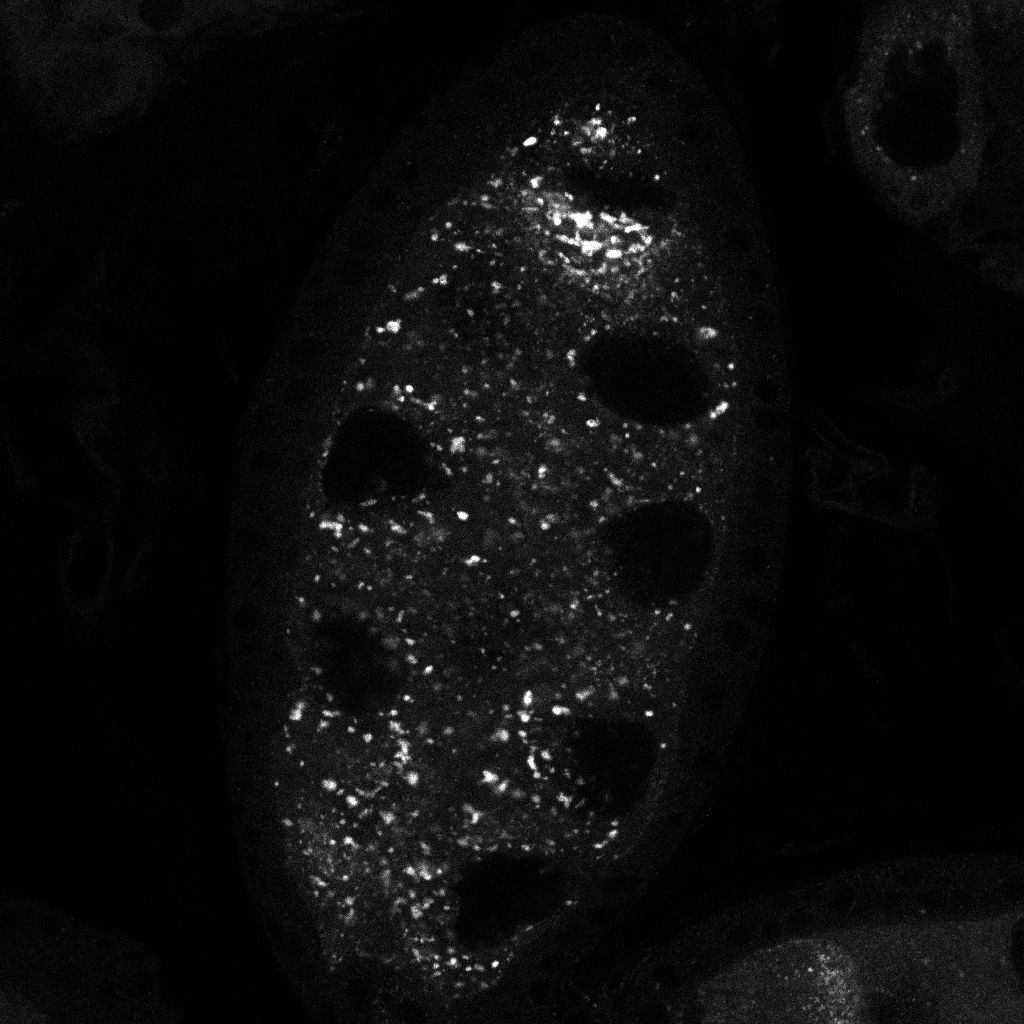

Supplement: Supplementary file 8 — Source data Fig. 5 [file 44319_2024_344_MOESM8_ESM.zip › Source Data Fig. 5/5K/K_sar1RNAi.tif]

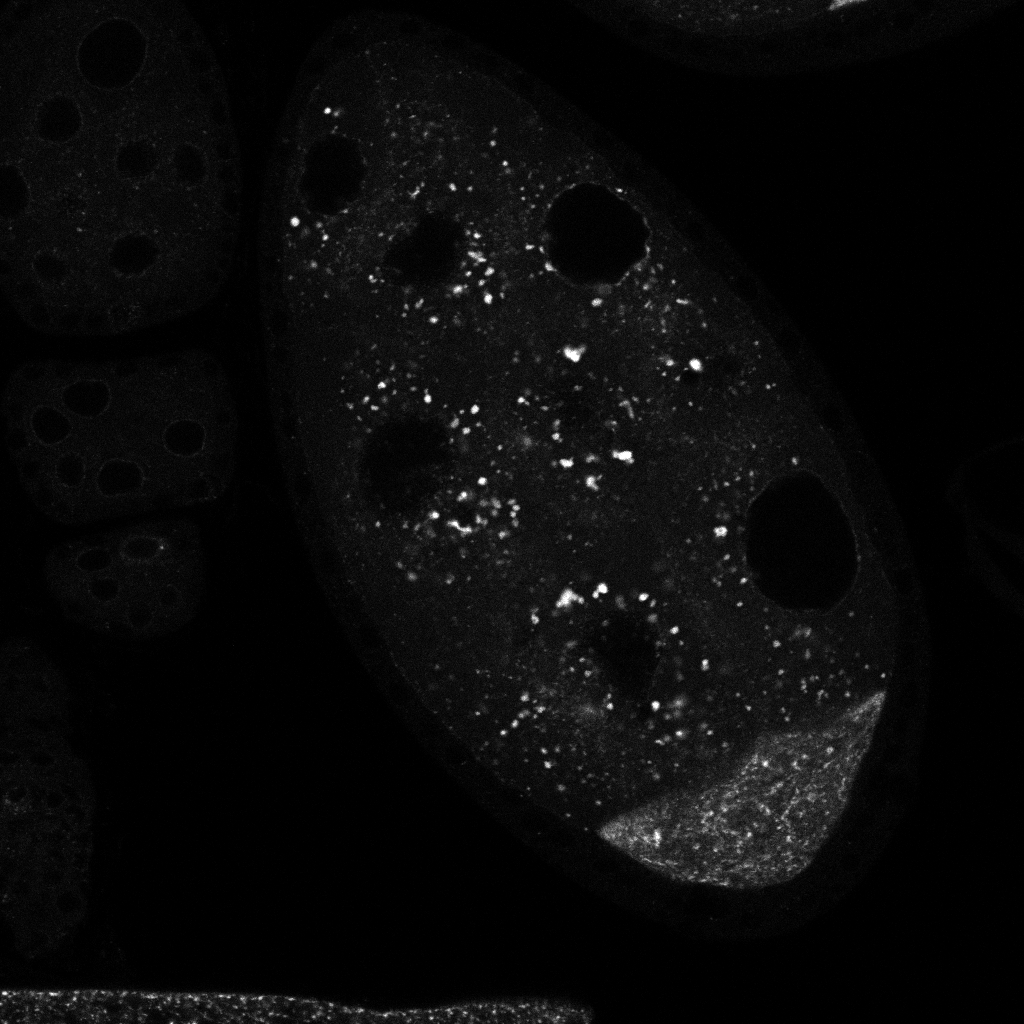

Supplement: Supplementary file 8 — Source data Fig. 5 [file 44319_2024_344_MOESM8_ESM.zip › Source Data Fig. 5/5K/K_mCherryRNAi.tif]

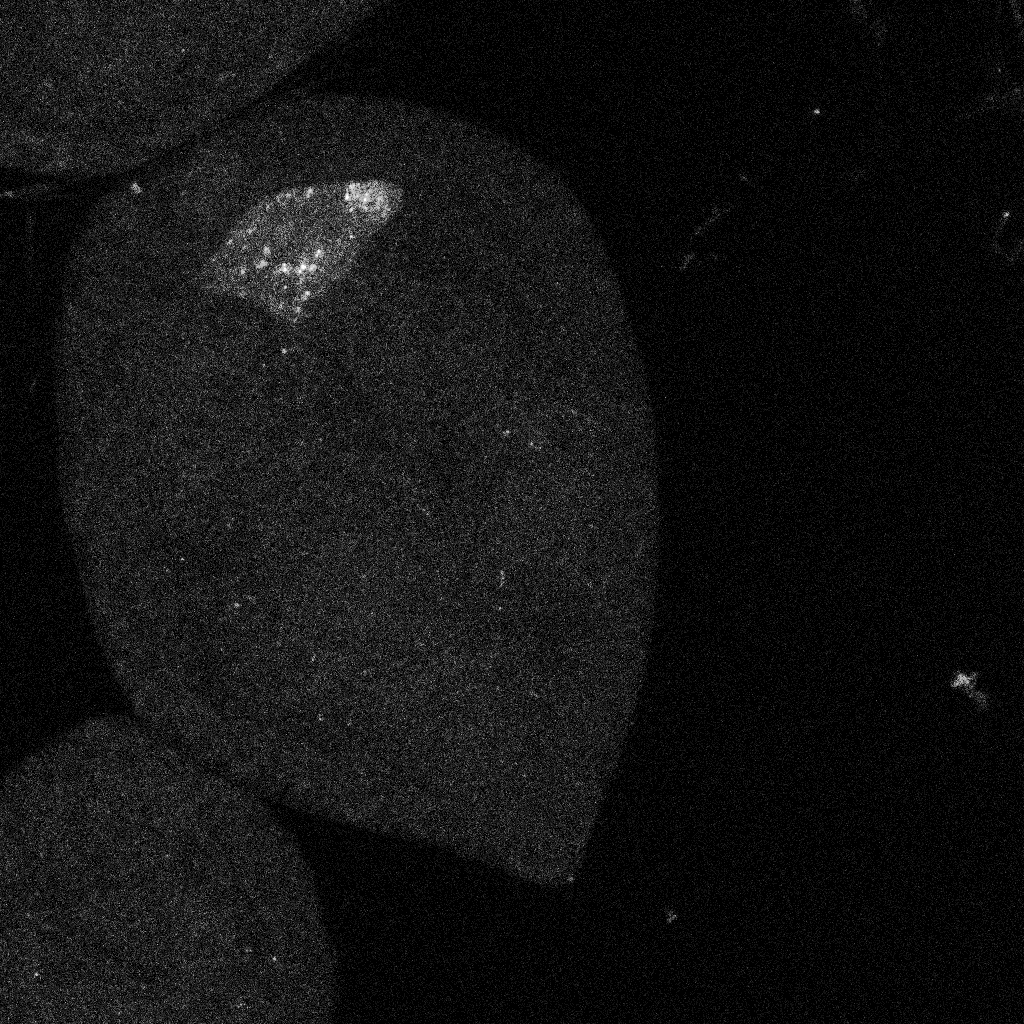

Supplement: Supplementary file 9 — Source data Fig. 6 [file 44319_2024_344_MOESM9_ESM.zip › Source Data Fig. 6/6A/A_sar1RNAi.tif]

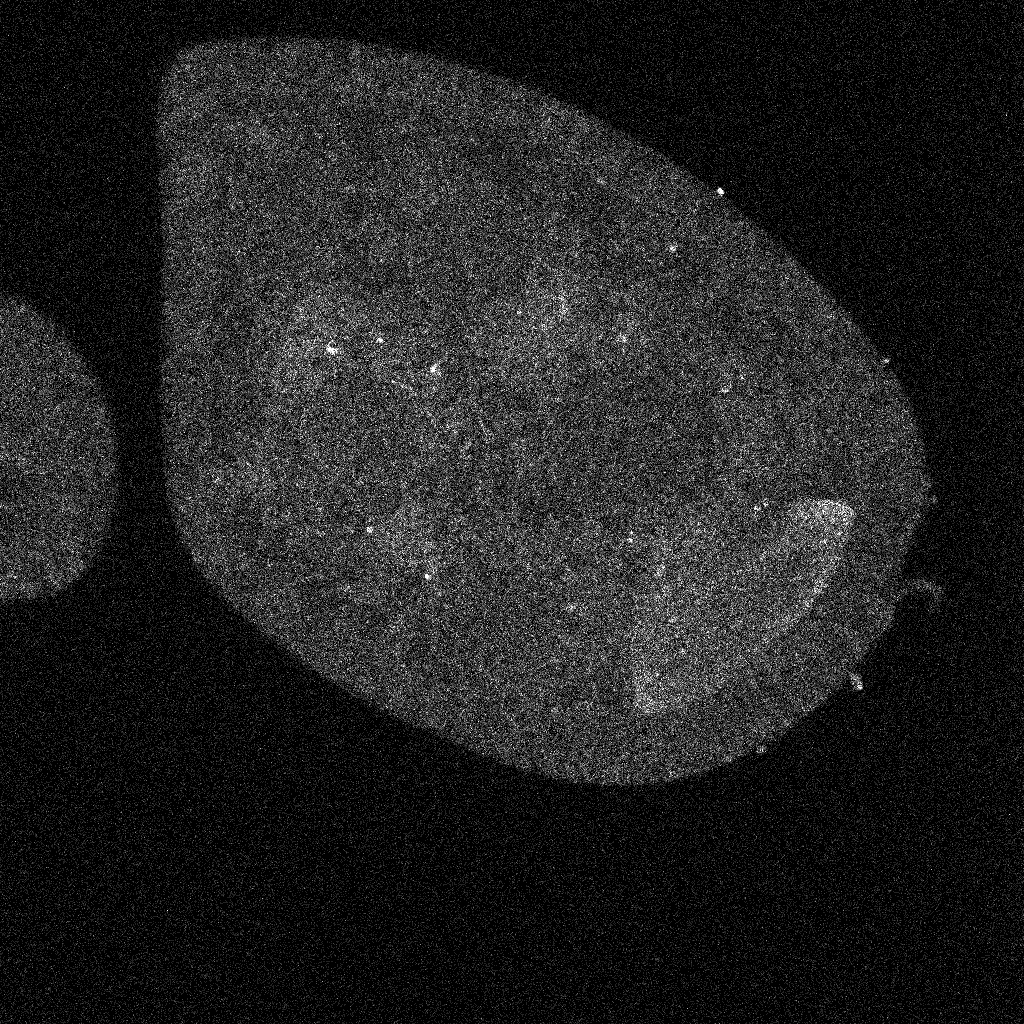

Supplement: Supplementary file 9 — Source data Fig. 6 [file 44319_2024_344_MOESM9_ESM.zip › Source Data Fig. 6/6A/A_mCherryRNAi.tif]

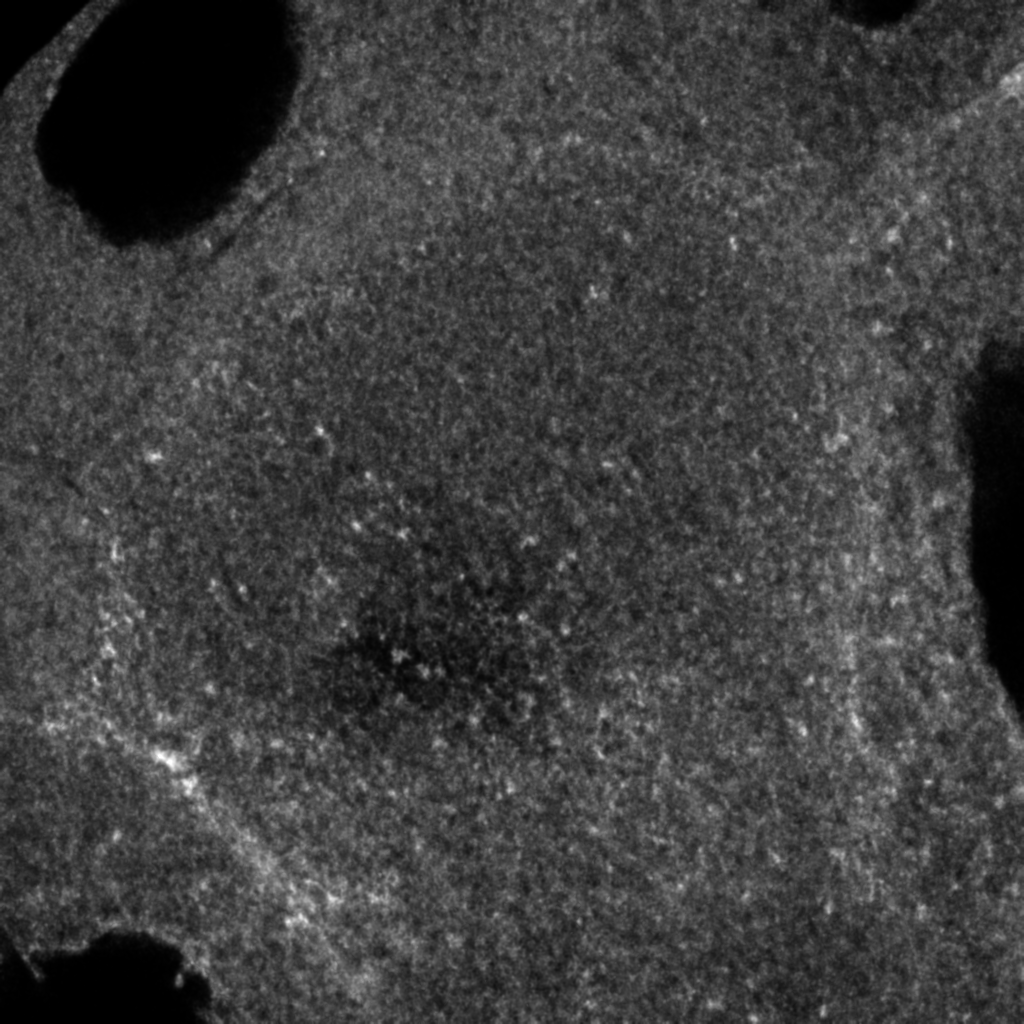

Supplement: Supplementary file 9 — Source data Fig. 6 [file 44319_2024_344_MOESM9_ESM.zip › Source Data Fig. 6/6E/E_sar1RNAi.tif]

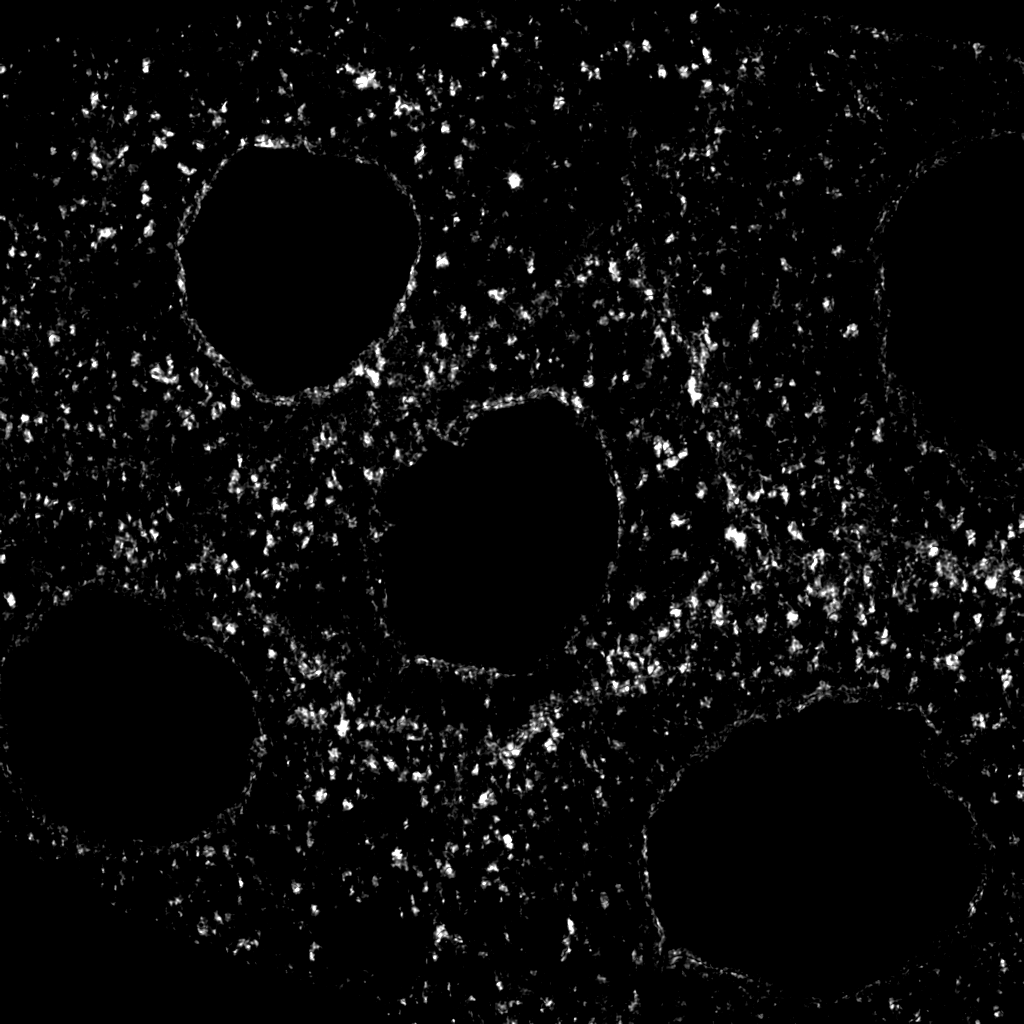

Supplement: Supplementary file 9 — Source data Fig. 6 [file 44319_2024_344_MOESM9_ESM.zip › Source Data Fig. 6/6E/E_mCherryRNAi.tif]

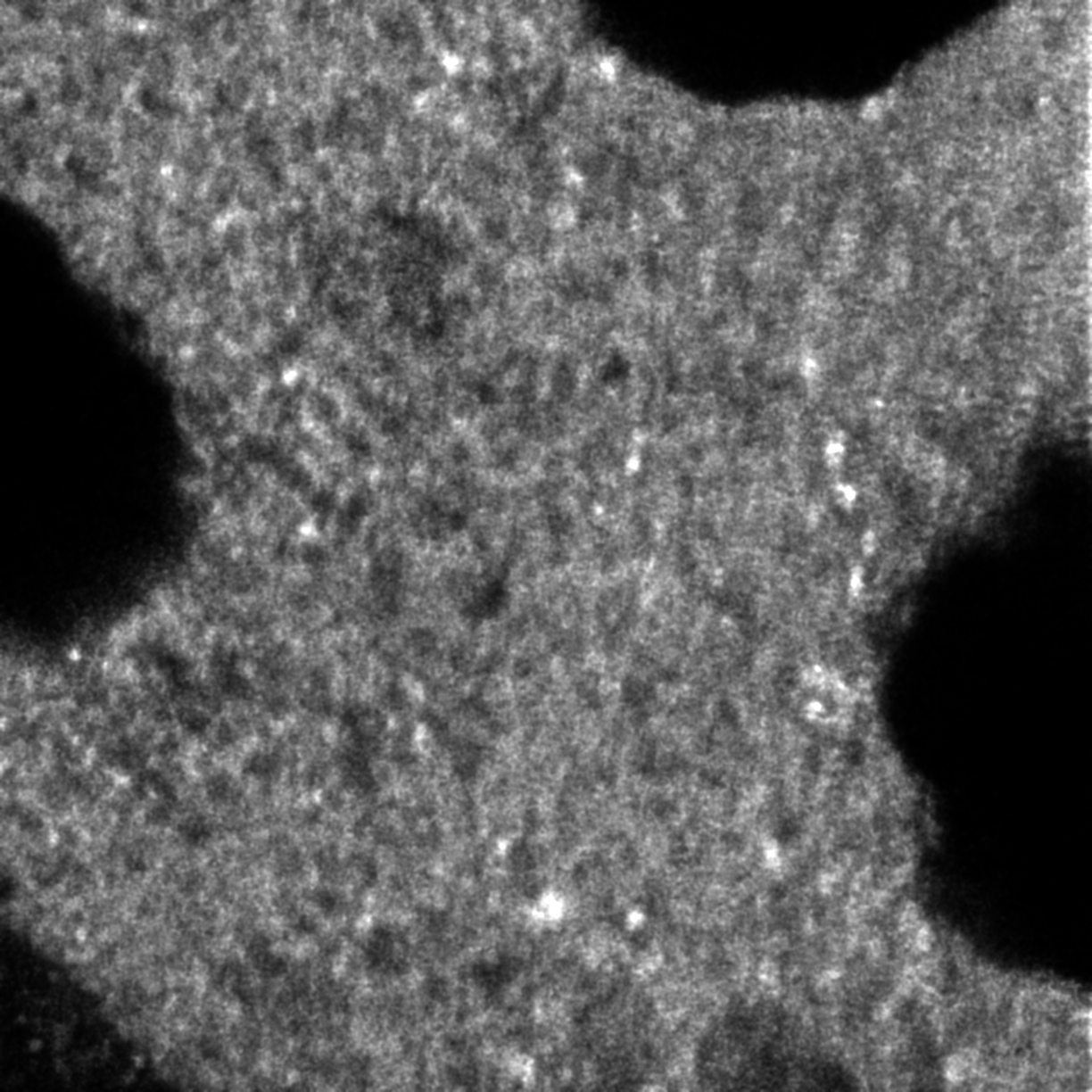

Supplement: Supplementary file 9 — Source data Fig. 6 [file 44319_2024_344_MOESM9_ESM.zip › Source Data Fig. 6/6H/H_sar1RNAi.tif]

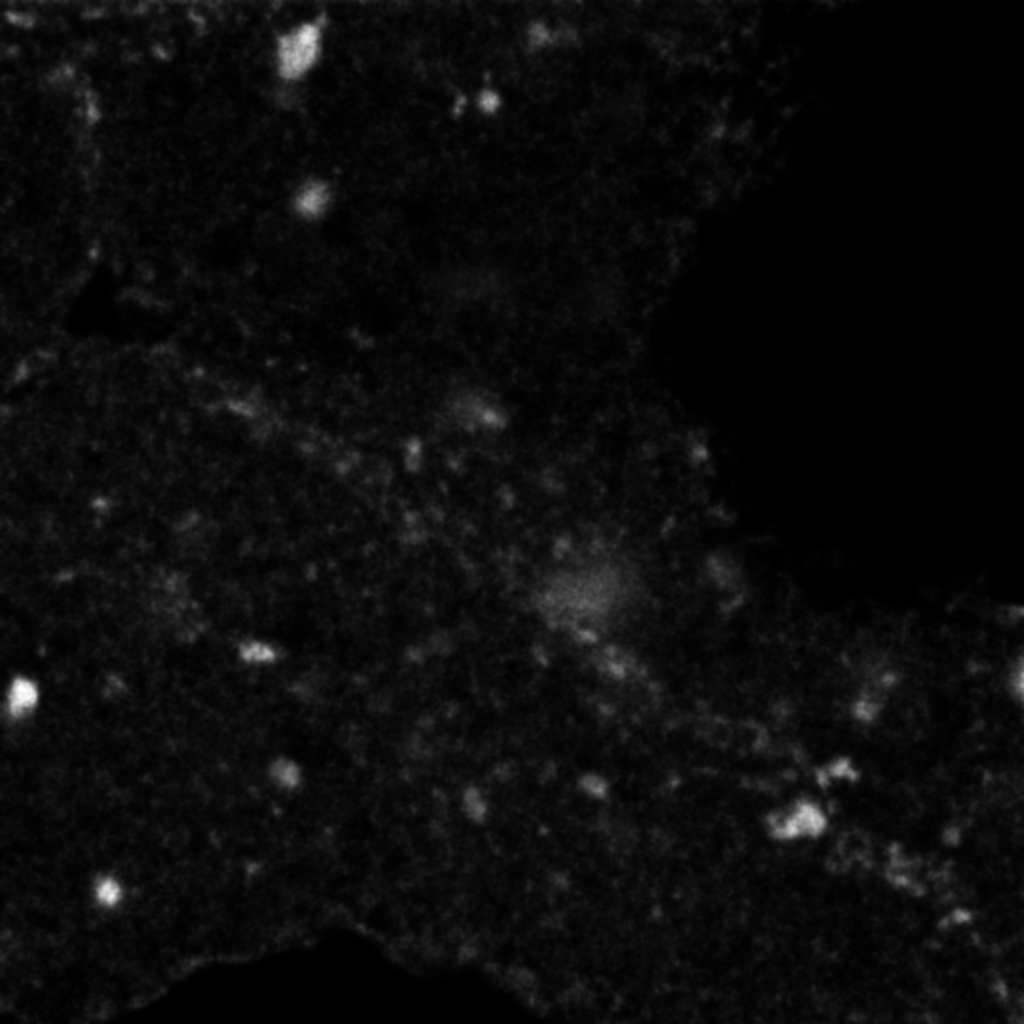

Supplement: Supplementary file 9 — Source data Fig. 6 [file 44319_2024_344_MOESM9_ESM.zip › Source Data Fig. 6/6H/H_mCherryRNAi.tif]
