# Supplementary material for: The role of ER exit sites in maintaining P-body organization and integrity during Drosophila melanogaster oogenesis
Source: EMBO Rep. 2024 Dec 9;26(2):494–520. doi: 10.1038/s44319-024-00344-x (PMC11772875; doi:10.1038/s44319-024-00344-x)
Supplement: Supplementary file 10 — Expanded View Figures [file 44319_2024_344_MOESM10_ESM.pdf]

Expanded View Figures

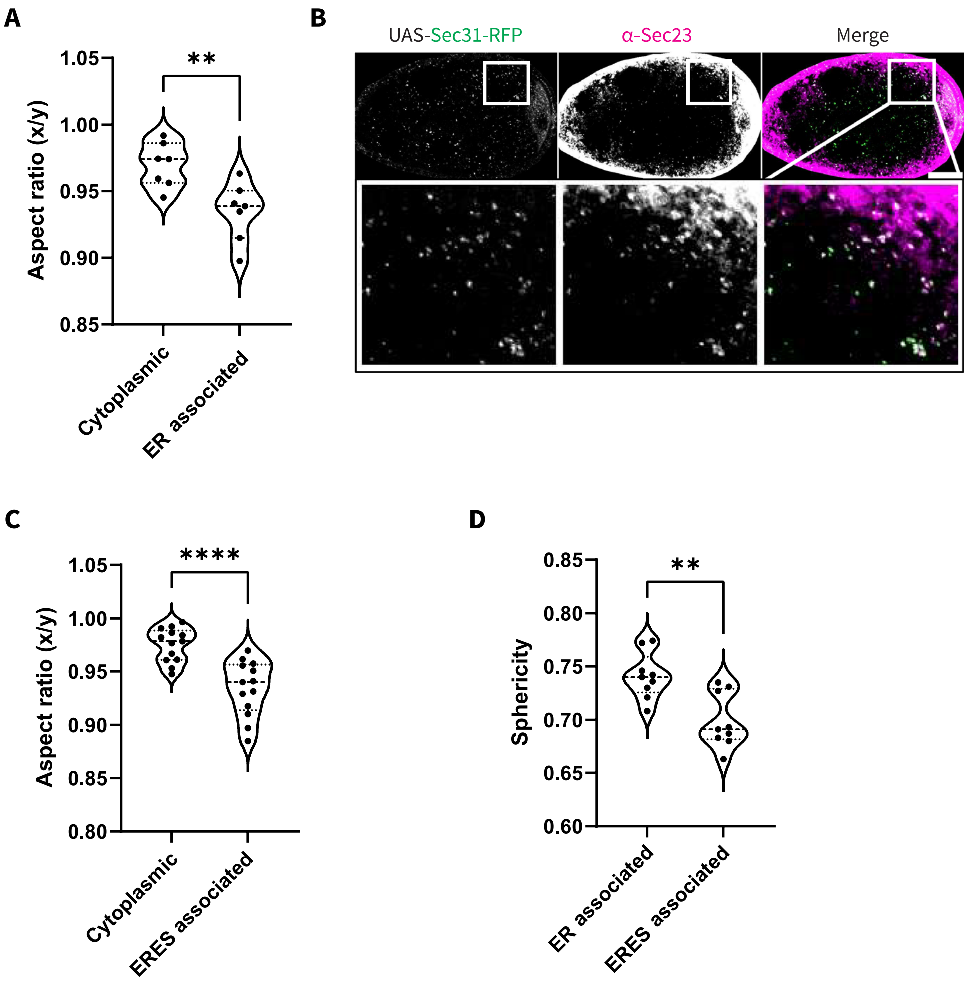

Figure EV1. P-bodies colocalized with ER exit sites are distinct from cytoplasmic P-bodies.

(relating to Fig. 1). (**A**) Aspect ratio of cytoplasmic and ER-associated P-bodies ($n = 7$, biological replicates). $P = 0.0070$. (**B**) Co-visualization of Sec31-RFP with Sec23. XY projections of 5 optical Z slices of 0.3 µm. Scale bars are 20 µm and 3.3 µm, respectively, in zoomed inset. (**C**) Aspect ratio of cytoplasmic and ERES-associated P-bodies ($n = 13$, biological replicates). $P < 0.0001$. (**D**) Sphericity measurement comparing ER and ERES-associated P-bodies ($n = 9$, biological replicates). $P = 0.0028$. Significance calculated with Mann–Whitney statistical test. Error bars represent standard deviation. ****$P < 0.0001$.

 © The Author(s)

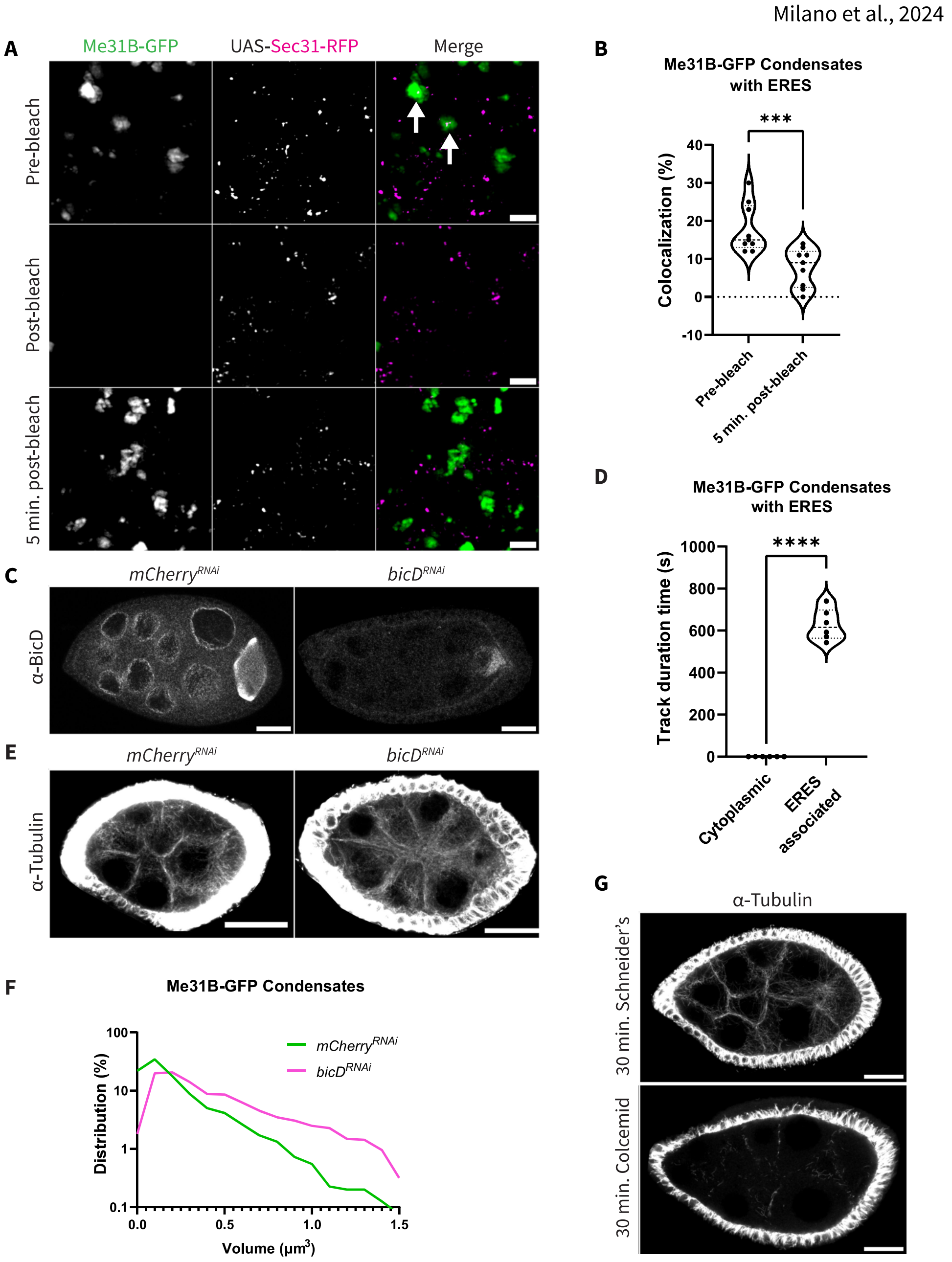

Figure EV2. ERES-associated P-bodies are dynamically distinct from cytoplasmic P-bodies and are removed from ERES-associated P-bodies by the microtubule network.

(relating to Figs. 2 and 3). (**A**) Co-visualization of Me31B-GFP and Sec31-RFP upon fluorescence recovery after photobleaching. Post-bleach image was acquired immediately after photobleaching. White arrows show ERES-associated P-bodies. Scale bar is 1 μm. (**B**) Colocalization analysis of Me31B-GFP condensates with Sec31-RFP-labeled ERES ($n = 9$, biological replicates). $P = 0.0014$. Significance calculated with a Mann–Whitney statistical test. (**C**) BicD expression in *mCherry*^{RNAi} and *bicD*^{RNAi} egg chambers. (**D**) Track duration analysis of Me31B-GFP condensates associated with ERES ($n = 6$, biological replicates). $P < 0.0001$. Significance calculated with a t-test. (**E**) α-Tubulin labeled microtubules in *mCherry*^{RNAi} and *bicD*^{RNAi} egg chambers. (**F**) Me31B-GFP condensate volume distribution in *mCherry*^{RNAi} and *bicD*^{RNAi} backgrounds. X-axis is shown on a log scale. (**G**) Microtubule integrity in egg chambers after 30 mins incubation in Schneider's media or 10 mM colcemid solution. All images are XY projections of 5 optical Z slices of 0.3 μm. Scale bars are 20 μm. Error bars represent standard deviation. ****$P < 0.0001$.

© The Author(s)

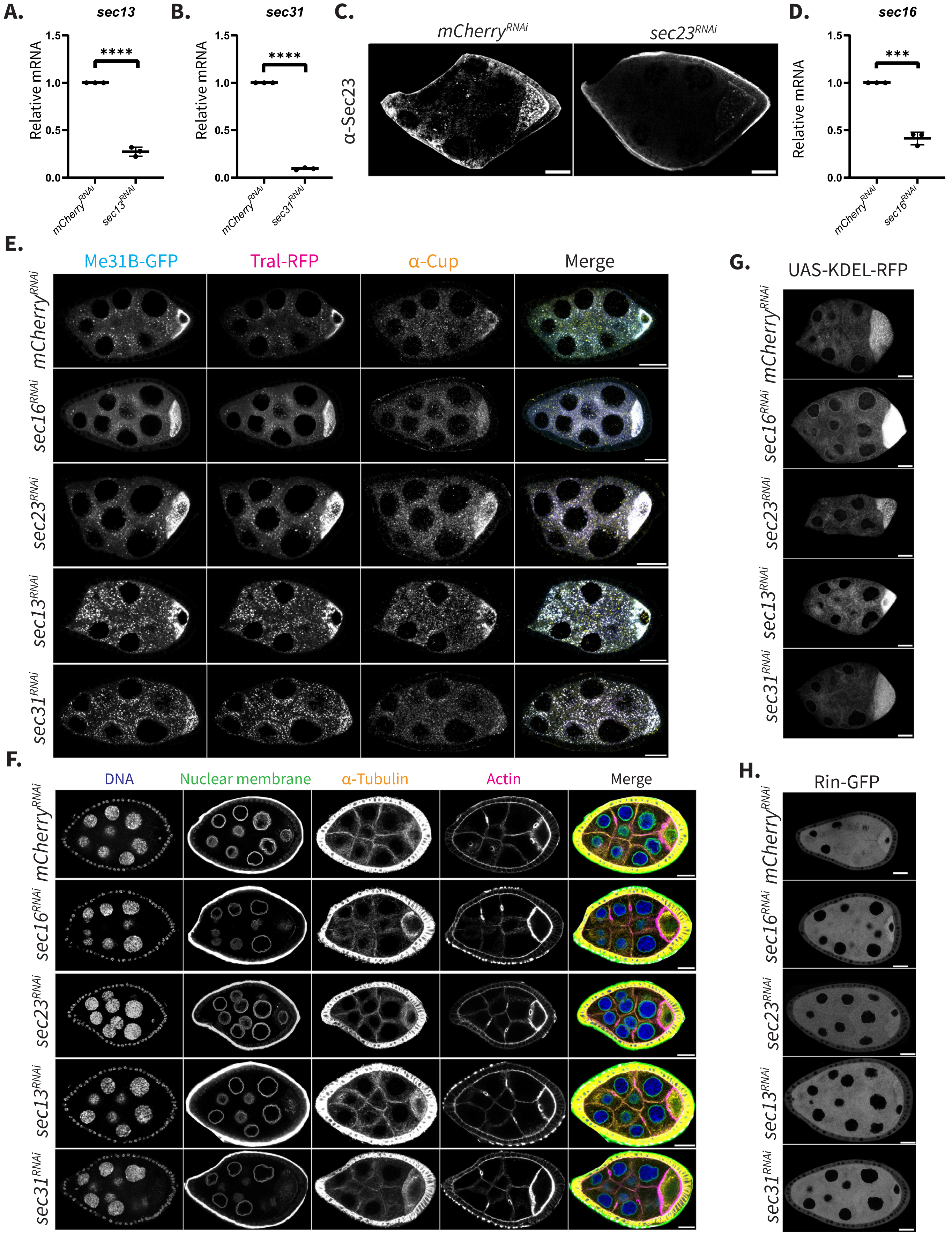
A.
sec13
Relative mRNA
****
mCherry^RNAi
sec13^RNAi
B.
sec31
Relative mRNA
****
mCherry^RNAi
sec31^RNAi
C.
mCherry^RNAi
sec23^RNAi
α-Sec23
D.
sec16
Relative mRNA
***
mCherry^RNAi
sec16^RNAi
E.
Me31B-GFP
Tral-RFP
α-Cup
Merge
mCherry^RNAi
sec16^RNAi
sec23^RNAi
sec13^RNAi
sec31^RNAi
F.
DNA
Nuclear membrane
α-Tubulin
Actin
Merge
G.
UAS-KDEL-RFP
H.
Rin-GFP

Figure EV3. COPII vesicle proteins affect the organization of putative P-body protein condensates.

(relating to Fig. 4). (**A**, **B**) Assessing knockdown efficiency of each ERES component via RT-qPCR. ($n = 3$, biological replicates). For *sec13*^{RNAi} and *sec31*^{RNAi}, $P < 0.0001$. (**C**) Immuno-detection of Sec23 in *mCherry*^{RNAi} and *sec23*^{RNAi} egg chambers. (**D**) Assessing knockdown efficiency of *sec16*^{RNAi} via RT-qPCR, $P = 0.0001$. (**E**) Full egg chamber images of Fig. 3B. (**F**) DNA (DAPI), nuclear membranes (wheat germ agglutinin), microtubules (α-Tubulin), and actin (phalloidin) in COPII component knockdown backgrounds. (**G**) KDEL-RFP visualized in knockdown nurse cells of each ERES component. (**H**) Rin-GFP visualized in each ERES component knockdown background. All images are XY projections of 5 optical Z slices of 0.3 µm. Scale bars are 20 µm. Significance calculated with a t-test. Error bars represent standard deviation. ****$P < 0.0001$.

© The Author(s)

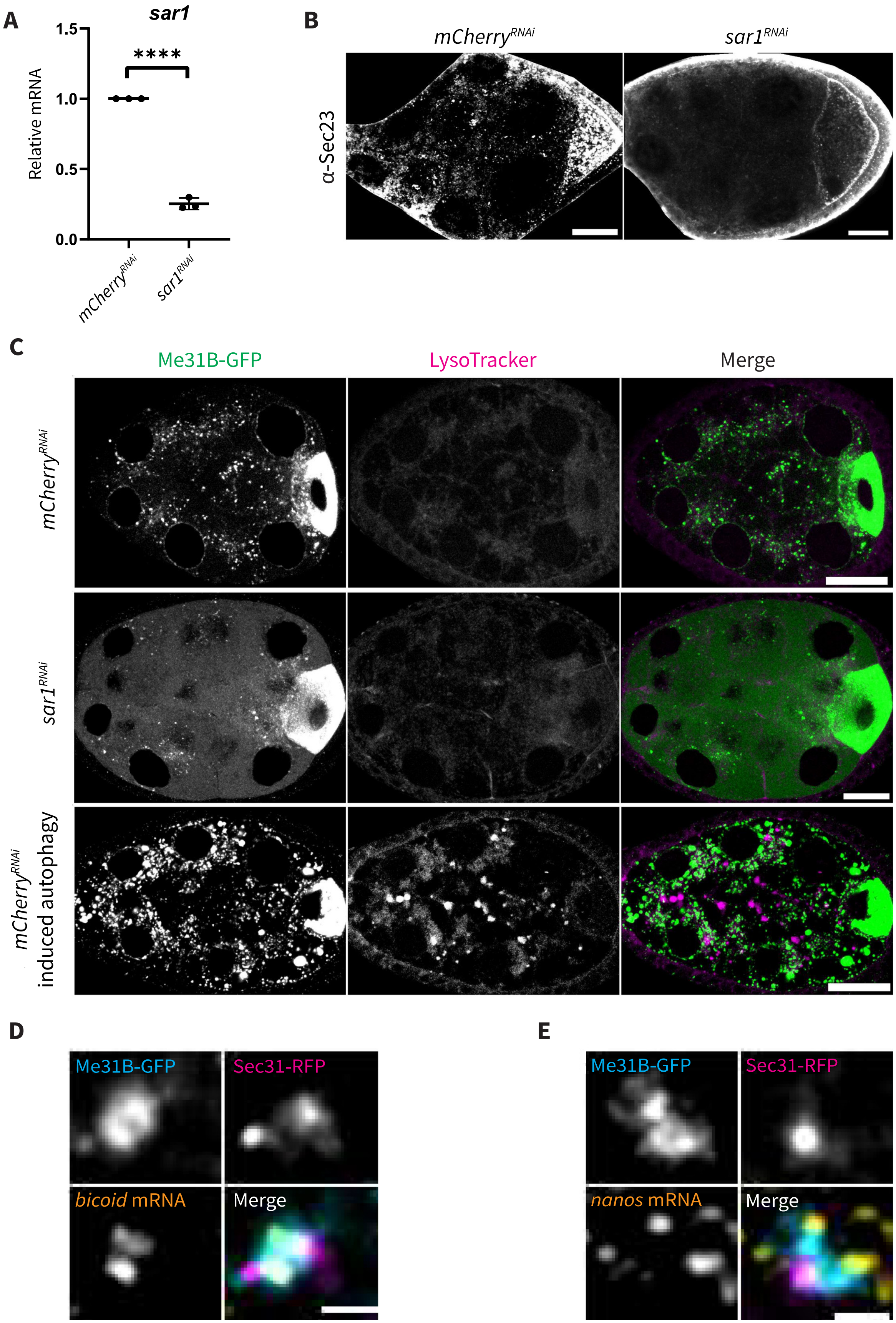
A
sar1
1.5
1.0
0.5
0.0
Relative mRNA
****
mCherry^RNAi
sar1^RNAi
B
mCherry^RNAi
sar1^RNAi
α-Sec23
C
Me31B-GFP
LysoTracker
Merge
mCherry^RNAi
sar1^RNAi
mCherry^RNAi induced autophagy
D
Me31B-GFP
Sec31-RFP
bicoid mRNA
Merge
E
Me31B-GFP
Sec31-RFP
nanos mRNA
Merge

 © The Author(s)

Figure EV4. In the absence of ER exit sites, P-body integrity is compromised.

(relating to Fig. 5). (**A**) Relative levels of *sar1* mRNA in *sar1*^{RNAi} egg chambers detected with RT-qPCR. ($n = 3$, biological replicates). $P < 0.0001$. Significance calculated with a t-test. Error bars represent standard deviation from the mean represented by the center bar. ****$P < 0.0001$. (**B**) Sec23 visualized in *mCherry*^{RNAi} and *sar1*^{RNAi} egg chambers. Scale bars are 20 µm. (**C**) Co-visualization of Me31B-GFP and LysoTracker in *mCherry*^{RNAi} and *sar1*^{RNAi} egg chambers and in the background of induced autophagy. Scale bars are 20 µm. (**D**, **E**) Me31B-GFP, Sec31-RFP, and *bicoid* mRNA or *nanos* mRNA visualized with smFISH probes. Scale bars are 1 µm. All images are XY projections of 5 optical Z slices of 0.3 µm.

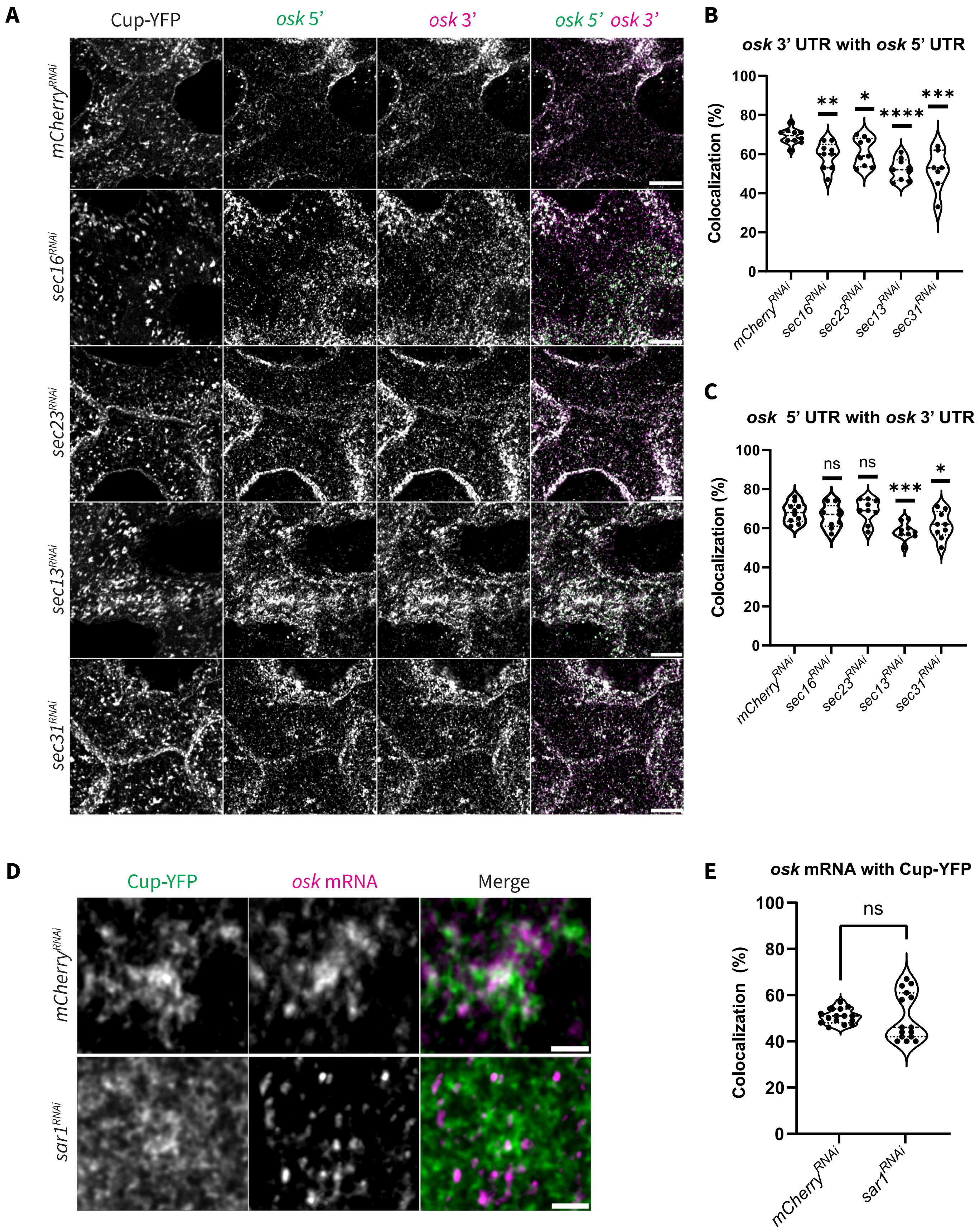
A
Cup-YFP
osk 5'
osk 3'
osk 5' osk 3'
mCherry^RNAi
sec16^RNAi
sec23^RNAi
sec13^RNAi
sec31^RNAi
B
osk 3' UTR with osk 5' UTR
Colocalization (%)
100
80
60
40
20
0
**
*
****
***
mCherry^RNAi
sec16^RNAi
sec23^RNAi
sec13^RNAi
sec31^RNAi
C
osk 5' UTR with osk 3' UTR
Colocalization (%)
ns
ns
***
*
D
Cup-YFP
osk mRNA
Merge
mCherry^RNAi
sar1^RNAi
E
osk mRNA with Cup-YFP
Colocalization (%)
ns
mCherry^RNAi
sar1^RNAi

 © The Author(s)

Figure EV5. Sar1 knockdown leads to attenuation of P-body function.

(relating to Fig. 6). (**A**) Cup-YFP, *oskar* 5′ UTR, and *oskar* 3′ UTR visualized with respective smFISH probes in each of the COPII component knockdown background. XY projections of 5 optical Z slices of 0.3 μm. Scale bars are 20 μm. (**B**) Colocalization analysis of *oskar* 3′ UTR with *oskar* 5′ UTR ($n = 10$, biological replicates). For $sec16^{RNAi}$, $P = 0.0010$. For $sec23^{RNAi}$, $P = 0.0104$. For $sec13^{RNAi}$, $P < 0.0001$. For $sec31^{RNAi}$, $P = 0.0003$. (**C**) Colocalization analysis of *oskar* 5′ UTR with *oskar* 3′ UTR ($n = 10$, biological replicates). For $sec16^{RNAi}$ and $sec23^{RNAi}$, P = n.s. For $sec13^{RNAi}$, $P = 0.0006$. For $sec31^{RNAi}$, $P = 0.0451$. (**D**) STED images of a single P-body labeled with Cup-YFP and *oskar* mRNA. XY projections of 3 optical Z slices of 0.22 μm. Scale bars are 2 μm. (**E**) Colocalization analysis of *oskar* mRNA with Cup-YFP ($n = 15$, biological replicates). P = n.s. Significance calculated with a Mann–Whitney statistical test. Error bars represent standard deviation from the mean represented by the center bar. ****$P < 0.0001$.

© The Author(s)
